# Percutaneous acetabuloplasty for metastatic acetabular lesions

**DOI:** 10.1186/1471-2474-9-66

**Published:** 2008-05-05

**Authors:** Giulio Maccauro, Francesco Liuzza, Laura Scaramuzzo, Alessandro Milani, Francesco Muratori, Barbara Rossi, Victor Waide, Giandomenico Logroscino, Carlo A Logroscino, Nicola Maffulli

**Affiliations:** 1 Department of Orthopaedics and Traumatology Catholic University. Agostino Gemelli Hospital – Rome, Italy; 2 Department of Orthopaedic and Traumatology Aurelia Hospital – Rome, Italy; 3 Institute of "Patologia Medica" Catholic University. Agostino Gemelli Hospital – Rome, Italy; 4 San Pietro Fatebenefratelli Hospital – Rome, Italy; 5 Manager Stryker, Italy; 6 Department of Orthopaedics and Traumatology Keele University School of Medicine – Staffordshire, UK

## Abstract

**Background:**

Osteolytic metastases around the acetabulum are frequent in tumour patients, and may cause intense and drug-resistant pain of the hip. These lesions also cause structural weakening of the pelvis, limping, and poor quality of life. Percutaneous acetabuloplasty is a mini-invasive procedure for the management of metastatic lesions due to carcinoma of the acetabulum performed in patients who cannot tolerate major surgery, or in patients towards whom radiotherapy had already proved ineffective.

**Methods:**

We report a retrospective study in 25 such patients (30 acetabuli) who were evaluated before and after percutaneous acetabuloplasty, with regard to pain, mobility of the hip joint, use of analgesics, by means of evaluation forms: Visual Analog Scale, Harris Hip Score, Western Ontario and McMaster Universities Index of Osteoarthritis (WOMAC), Eastern Cooperative Oncology Group (ECOG). The results obtained were analysed using the χ^2 ^Test and Fisher's exact test. Significance was sent at P < 0.05.

**Results:**

Marked clinical improvement was observed in all patients during the first six post-operative months, with gradual a worsening thereafter from deterioration of their general condition.

Complete pain relief was achieved in 15 of our 25 (59%) of patients, and pain reduction was achieved in the remaining 10 (41%) patients. The mean duration of pain relief was 7.3 months. Pain recurred in three patients (12%) between 2 weeks to 3 months. No major complications occurred. There was transient local pain in most cases, and 2 cases of venous injection of cement without clinical consequences.

**Conclusion:**

Percutaneous acetabuloplasty is effective in improving the quality of life of patients with osteolytic bone tumours, even though the improvement is observed during the first 6 months only. It can be an effective aid to chemo- and radiotherapy in the management of acetabular metastases.

## Background

Osteolytic metastases around the acetabulum are frequent in tumour patients, and may cause intense and drug-resistant pain of the hip. These lesions also cause structural weakening of the pelvis, limping, and poor quality of life. Such patients may require walking aids, and are often wheelchair-bound. The osteolytic lesions often lead to pathological fractures, forcing the patients to bed rest, with a considerable increase of co-morbidity.

Radiotherapy alone is usually unable to control the pain and/or to restore the integrity of the acetabular area, so as to allow a return to early weight-bearing [1,2]. Acetabular reconstruction is invasive, and carries a high rate of local and systemic complications in patients with multiple metastases [3,4]. Acrylic cement has been used to fill secondary benign or malignant osteolytic lesions of the long bones after curettage [5,6]. Vertebroplasty and kyphoplasty are able to obtain reduction or the elimination of pain by injection of acrylic cement into the pathological or osteoporotic fractures of the spine [7-10]. Similar techniques have been reported in the management of metastatic lesions around the acetabulum [11,12]. We report the results of a retrospective study on 25 patients with acetabular metastases, who received percutaneous acetabuloplasty.

## Methods

### Patients

In the period January 2004 to March 2007, 25 patients (30 acetabular lesions) gave their written informed consent to undergo percutaneous acetabuloplasty. All patients presenting with metastatic acetabular disease were evaluated by a multidisciplinary team, which included oncologists, radiotherapists, orthopaedic surgeons and radiologists. Patients received the procedure if it was not possible to perform surgical reconstruction of the acetabulum. The inclusion and exclusion criteria are reported in Table 1 and 2(a, b).

**Table 1:** Indications to acetabuloplasty

Weight-bearing acetabular osteolisis
Hip pain resistant to drugs
Patients with multiple metastases
Short life expectancy
Inability to tolerate major surgery
Histotype differing from that of the kidney and the thyroid gland
Radioterapy ineffectiveness

**Table 2:** 

a: Absolute contraindications to acetabuloplasty
Acetabular fracture
Pelvic discontinuity

b: Relative contraindications to acetabuloplasty

Radiographic signs of medial wall interruption
Local infection
Hemorrhagic disorders

All patients were evaluated before and 1, 3, 6, 12 months after acetabuloplasty with regard to pain, hip joint mobility, use of analgesics using the following criteria Visual Analog Scale, Harris Hip Score, Western Ontario and McMaster Universities Index of Osteoarthritis (WOMAC), Eastern Cooperative Oncology Group (ECOG). Also the type and quantity of drugs taken by the patients to control the pain, during the preoperative and postoperative periods, were recorded.

**Figure 1:**
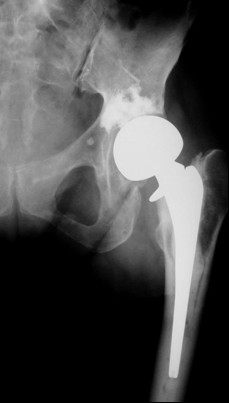
Illustrative images of patients with fracture of the femoral neck and acetabular osteolysis managed with bipolar endoprosthesis and acetabuloplasty.

**Figure 2:**
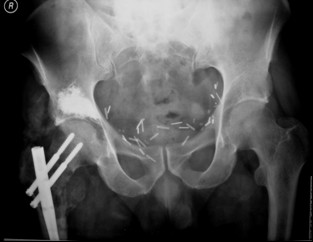
Femoral and acetabular osteolysis treated with a locked intramedullary reconstruction nail and acetabuloplasty.

**Figure 3:**
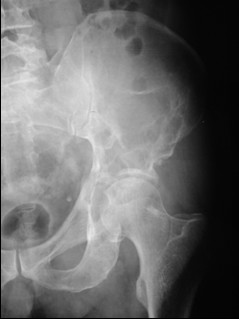
Preoperative plain radiographs showing wide acetabular osteolysis.

**Figure 4:**
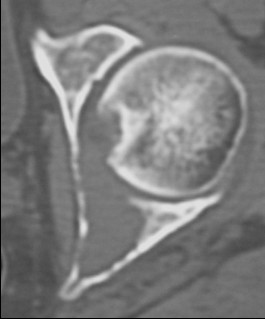
CT.

**Figure 5:**
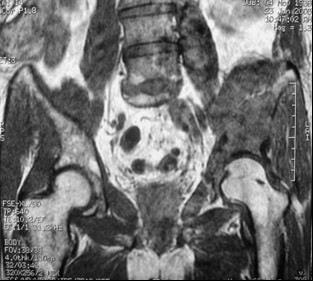
MRI of the corresponding area.

**Figure 6:**
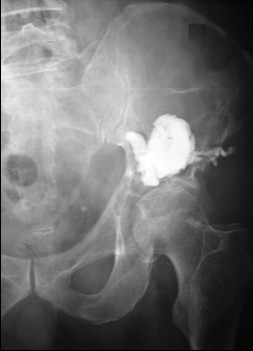
Postoperative plain radiographs.

**Figure 7:**
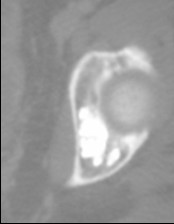
CT showing filling of the acetabular lesion.

Twenty-five patients (30 acetabuli: 11 males and 14 female; average age 59.9 years; left side affected in 17 instances, right side affected in 13 instances) underwent the index procedure. At the time of percutaneous acetabuloplasty, nine biopsies were performed using the same set of instruments (Stryker Corp. Kalamazoo, MI, USA). In two patients, together with acetabuloplasty, a bipolar hemiarthroplasty was implanted for a transcervical fracture of the femoral neck (fig 1). In three other patients, a reconstruction intramedullary locked nail was implanted for a fracture or an impending fracture of the femoral diaphysis (fig 2). The primary tumour originated from the breast (15 patients), lung (8 patients), prostate (2 patients). The presence of an osteolytic acetabular lesion was evaluated preoperatively by standard radiography, CT and MRI. (fig 3, 4, 5). Postoperatively standard radiography and CT were performed (fig 6, 7)

### Operative technique

The procedure was performed under general (n = 7 patients), spinal (n = 15 patients) or local anaesthesia (n = 3 patients). Patients are positioned on a fracture table either supine or in lateral decubitus, and antero-lateral and postero-lateral portals are used to fill the whole cavity with cement. After having prepared a sterile field, two Kirschner wires are inserted into the acetabulum under image intensifier control. Using the two Kirschner wires as an initial guide, two appropriately sized introducer cannulae are slid over these Kirschner wires for cement injection (e.g. 10 Gauge), and inserted into the osteolytic cavity. The Kirschner wires are removed, and the cannula may be oriented with its bevelled stylet for subsequent cement injection in the desired location. If desired, prior to injecting the cement, a percutaneous bone biopsy can be taken using another cannula in conjunction with the introducer cannula (Stryker Bone Byopsy Kit, Stryker Corp Kalamazoo, MI, USA). This allows to obtain a small cylindrical sample of tissue. The positioning of the cannula and the injection of the cement are constantly monitored in antero-posterior and oblique projections. While the cement is being injected, no leakage should occur outside of the bone. Should this be observed, the cement injection should be stopped, and the cannula may have to be withdrawn, so as to allow the cement that has already been injected to harden, and to avoid any further leakage. At a later stage, more cement can be injected, if necessary. Some authors suggest to inject contrast medium before using the cement, to see how this will spread inside the osteolytic lesion [13]. This was not performed in this study. If the cement leaks in the hip joint, in addition to stopping the injection, the leg is brought to the whole range of motion, so as to spread the leaked cement over the surface of the joint before it hardens.

If the patients received general or spinal anaesthesia, they were positioned in lateral or supine decubitus. If local anaesthesia was used (10 ml of 7.5 mg of ropivacaine mixed with 10 ml of 1% mepivacaine, AstraZeneca S.p.A. – Italia,), the patient was supine. All procedures were performed by the same surgeon. Each patient received 2 g of intravenous cefazolin 30 minutes before surgery. The average length of time of the procedure was 30 minutes. The average hospital stay was 3 days.

### Statistics

Descriptive statistics were calculated. The results obtained were analysed using the χ^2 ^Test and Fisher's exact test. Singificance was sent at P < 0.05.

## Results

In all patients, mechanical stabilization of the osteolytic lesion was achieved, there were no pathological fractures, and no clinical symptoms related to cement leakage. Eight patients reported transiently increased pain of the hip and pyrexia in the immediate postoperative 48 hours. In two patients, the cement was injected in a vessel, but there were no clinical manifestations of this. The images were reviewed by a fellowship trained interventional radiologist who, in the absence of clinical symptoms, did not require any further imaging.

**Figure 8:**
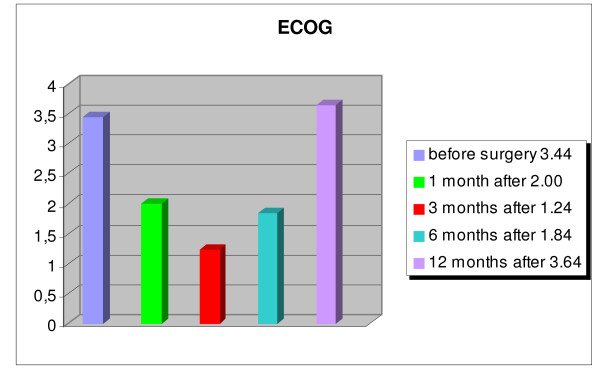
Eastern Cooperative Oncology Group (ECOG) values Patients: n = 25; p < 0.001.

**Figure 9:**
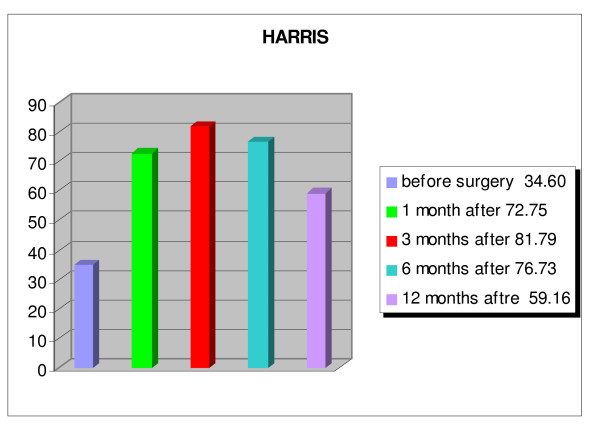
**Harris Hip score.** Patients: n = 25; p < 0.001.

**Figure 10:**
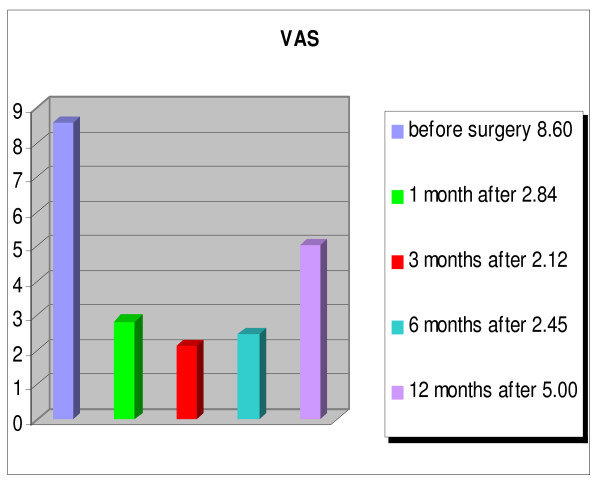
**Visual Analogue Pain (VAS) scale for pain. **Patients: n = 25; p < 0.001.

**Figure 11:**
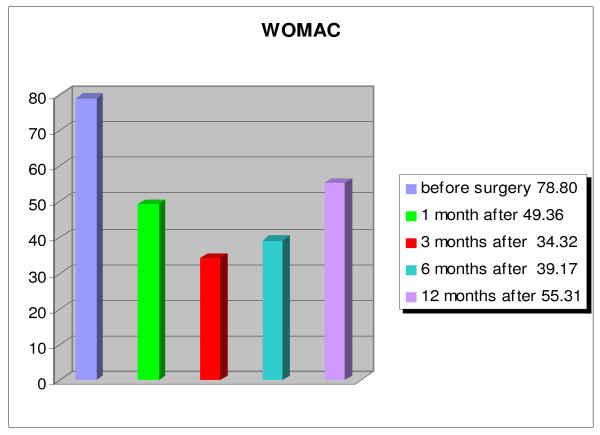
Patients: n = 25; p < 0.001.

An overall improvement of the quality of life of the patients was evident, and patients were able to return to their activities of daily living (fig. 8, 9, 10, 11). The WOMAC hip questionnaire showed marked local improvement. The ECOG index showed an improvement of the patients' general condition up to 6 months after the index procedure, with the average score improving from 3.44 preoperatively, to 2.00 at 1 month, 1.24 at 3 months, and 1.84 at 6 months. Worsening of the general condition was observed one year after the surgery, with an average score of 3.76, with 10 patients dead by that time. The HHS index showed an improvement in the ability to carry out standard daily activities autonomously, with a preoperative score of 34.60% rising to a postoperative score of 72.75% one month after the intervention, which further improved (81.79%) 3 months after the treatment. At the 1 year follow-up a worsening of the general condition was observed, with reduced autonomy, with the average HHS score dropping to 59.16%, yet still higher than the score recorded preoperatively. A similar trend was observed for pain, measured by the VAS score. Pain improved from an average preoperative score of 8.60 to 2.84 at 1 month, to 2.12 at 3 months, to 2.45 at 6 months, and to 5.06 at 1 year. The local function, evaluated by the WOMAC, showed a considerable improvement over the first 6 months after the treatment, from an average preoperative score of 78.80% to 39.17% at 6 months. One year after the injection, the local function dropped to an average score of 55.31%.

**Figure 12:**
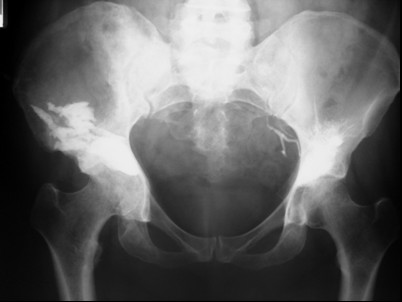
Bilateral metastatic osteolisys, showing vascular injection of the cement.

In summary, complete pain relief was achieved in 59% of patients (n = 15). Pain reduction was achieved in 10 of 25 patients (41%). The mean duration of pain relief was 7.3 months (median: 6 months). Pain recurred in three patients (12%) between 2 weeks to 3 months. Ten patients died, and 15 patients were still alive at the time of the one year follow up. The one-year survival rate was 40% (observation period: 1–30 months). No major complications occurred. There was transient local pain in most cases, and 2 cases of venous injection of cement without clinical consequences (fig. 12).

We observed a reduction or, in some patient, the cessation of analgesic drug assumption, compared to the preoperative period. It is impossible to quantify the reduction of analgesic drug assumption in patients with systemic neoplastic disease, due to the coexistence of multiple secondary lesions. Nevertheless, a marked reduction of analgesic drug assumption was observed in some (4 patients, 16%), even when radiotherapy was not effective.

## Discussion

Surgical reconstruction in metastatic disease of the acetabulum should fulfil three aims: resection of the tumour, filling of the bone defect, and stabilization of the skeletal segment. Usually, the lesion is curetted, followed by filling with cement and reinforcement with Steinman pins, or the use of a hip or pelvic prosthesis [3]. Although these procedures have a high rate of complications, and a death rate of about 50% within 12 months [4], the patient's life expectancy and the improvement of the quality of life produced justify the surgical risks.

Radiotherapy is indicated for bone pain caused either directly or indirectly by malignant lesions. Malignant lesions may produce pain from direct action on the nervous system [14]. However, different modalities of pain production may act in the presence of skeletal metastases (mild, moderate, mechanical) [15]. Injection of cement may immediately reduce pain in patients with acetabular metastases in the absence of protrusion or fracture, with at least partial pain relief in about 80% of patients [1,2,16,17] within two weeks of the injection. Radiotherapy does not improve the mechanical properties of the affected acetabular region, and transient osteoporosis is usually observed, with the risk of pathological fractures [1,2].

In 1995, Cotten [11] suggested to extend the vertebroplasty technique to the acetabulum for the management of secondary osteolytic lesions. Preliminary results showed that these techniques were effective as palliative methods for controlling pain and reinforcing bone. Acetabuloplasty uses a percutaneous injection of low viscosity acrylic cement into the osteolytic cavity of acetabulum. Acetabuloplasty aims to immediately restore the mechanical properties of the affected skeletal segment, it imparts increased resistance to compressive stresses to the treated acetabulum with prevention of continue microtrauma responsible of the increased risk of fractures, allowing immediate weight-bearing and preventing pathological fractures. It also reduces or eliminates pain. Furthermore, the exothermic reaction developed during the polymerization of the cement exerts a local cytotoxic action against the tumour. Acetabuloplasty is indicated in patients suffering from acetabular metastatic disease of the weight-bearing area, with drug-resistant pain of the hip, gait limitation and inability to tolerate major surgery, either from a local or systemic extension of the disease, or particularly poor clinical condition (fig. 3, 4, 5, 6, 7). Hip fractures are not a contraindication, and the opportunity to use a bipolar endoprosthesis combined with the injection of acetabular cement allows a mechanical support for the acetabulum. This enables the patient to avoid the risk of haemorrhage, and the problems linked to the preparation of the acetabulum. The same considerations can be made for combined lesions of the femoral shaft and the acetabulum, and for patients with bilateral disease. (fig. 1, 2, 12).

Acetabuloplasty can aid radiotherapy, both by improving pain and by providing a mechanical support at a stage during which radiotherapy alone would not be able to prevent pathological fractures. Complications may also occur in pelvic cementoplasty (Table 3), but, in our experience, they were only relatively minor.

**Table 3:** Known complications of acetabuloplasty

Post operative pain and fever (1–4 days)
Intrarticular injection
Vessel injection
Long-distance failure (progressive disease)
Renal failure

Following acetabuloplasty, our patients experienced marked pain relief and increased ability to walk, a return to normal daily and recreational activities, and an overall increase of quality of life. Acetabuloplasty seems to achieve the main goals of palliative management, namely improving clinical conditions using a low risk low cost procedure.

The mean duration of pain relief was 6 months. After that period, worsening of the patients' condition from progression of the disease influenced also the local results. At 1 year after acetabuloplasty, 10 patients had died from inevitable progression of the underlying conditions, 15 patients were still alive, with only one patient lost to follow-up. No major complications were observed.

To the best of the authors' knowledge, this present is the largest study to date. This retrospective study presents some limits. For this reason, prospective studies comparing cement injection with other minimally invasive techniques (chemoablation, cryoablation, thermal ablation) are desirable.

Following Cotten's experience, others have also used acetabuloplasty, obtaining above all an immediate improvement of the pain symptom after the injection, both in individual cases [13,18-21] and in larger series. Some have suggested a combined approach, with radiofrequency thermal ablation and percutaneous cementoplasty [22,23]. Toyota et al [22] reported on 17 adult patients with 23 painful bone metastases who underwent RF ablation therapy combined with cementoplasty over a 2-year period.

Schaefer [23] described a patient with a stage IV malignant melanoma and a pathological fracture of the left tibial plateau who underwent radiofrequency heat ablation and percutaneous cementoplasty for defect filling and stabilization. The exothermic reaction arising from the cement's polymerization is basically the same as that obtained by radiofrequency. Therefore, no increase of cytotoxic effect on the tumour should be obtained [23].

Acetabuloplasty is reliable for the management of acetabular osteolysis in patients who cannot be candidate to major surgery. In patients in whom radiotherapy was not effective, it can be performed by orthopaedic surgeons and interventional radiologists. In selected patients, local anaesthesia may be used. The contemporary presence of an impending or complete femoral fracture should be an indication for acetabular cementoplasty during the same anaesthesia.

This study cannot demonstrate that acetabuloplasty results in reduction of analgesic drugs in patients with multiple metastases, or whether the technique is superior to percutaneous radiofrequency. Nevertheless, bone cement is able to restore some of the compromised mechanical proprieties after filling bone cavities. Periacetabular defects may increase the vulnerability of the pelvis to fracture [24], depending on size and cortical involvement. Acetabular cement filling may lower the risk of periacetabular fractures, as little as 10% cement by volume could result in large compressive strength increases, thus reducing the risk of fractures. [25]

A randomized prospective study comparing the results between radiotherapy, radiofrequency and cementoplasty should be considered in the future.

## Conclusion

Percutaneous acetabuloplasty is a palliative mini-invasive technique that produces effective results in the management of acetabular osteolyses in patients with multiple metastases and with a low life expectancy. Percutaneous acetabuloplasty is effective in improving the quality of life of patients with osteolytic bone tumours, even though the improvement is observed during the first six months only. It can be an effective aid to chemo- and radiotherapy in the management of acetabular metastases.

## Competing interests

The authors declare that they have no competing interests.

## Authors' contributions

GM operated patients, conceived the study. FL drafted the manuscript. LS performed statistical analysis and participated in drafting. AM performed statistical analysis. FM participated in design of the study. BR followed patients. WV revised manuscript. GL followed patients. CAL coordinated group. NM participated in design and drafting and revised manuscript. All authors read and approved the final manuscript.

## Pre-publication history

The pre-publication history for this paper can be accessed here:


